# Attention-deficit/hyperactivity disorder (ADHD) in cultural context II: a comparison of the links between ADHD symptoms and waiting-related responses in Hong Kong and UK

**DOI:** 10.1007/s00787-024-02506-7

**Published:** 2024-06-27

**Authors:** Wendy W Y Chan, Kathy Kar-man Shum, Johnny Downs, Ngai Tsit Liu, Edmund J S Sonuga-Barke

**Affiliations:** 1 School of Academic Psychiatry, Institute of Psychiatry, Psychology & Neuroscience, King’s College London, London, UK; 2 Department of Psychology, The University of Hong Kong, Hong Kong, China; 3 Department of Child & Adolescent Psychiatry, Aarhus University, Aarhus, Denmark; 4 Department of Child & Adolescent Psychiatry, Institute of Psychiatry, Psychology & Neuroscience, DeCrespigny Park, SE5 8AF UK

**Keywords:** Waiting, Attention-deficit/hyperactivity disorder (ADHD), Delay aversion, Preschoolers, self-regulation, cross-cultural

## Abstract

The concept of attention-deficit/hyperactivity disorder (ADHD) is considered to have cross-cultural validity, but direct comparisons of its psychological characteristics across cultures are limited. This study investigates whether preschool children’s ADHD symptoms expressed in two cultures with different views about child behaviour and parenting, Hong Kong and the UK, show the same pattern of associations with their waiting-related abilities and reactions, an important marker of early self-regulation. A community sample of 112 preschoolers (mean age = 46.22 months; 55 from UK, 57 from HK) completed three tasks measuring different waiting elements – *waiting for rewards*, *choosing the amount of time to wait*, and *having to wait unexpectedly when a task is interrupted*. Participants’ waiting-related behavioural and emotional reactions were coded. Parents rated their children’s ADHD symptoms and delay aversion. Our findings revealed that the associations between ADHD symptoms and waiting-related responses were comparable in both UK and HK samples. This suggests that the core psychological characteristics of ADHD, particularly in relation to waiting behaviours, may exhibit similarity across cultural contexts. Future research can extend this cross-cultural analysis to other ADHD-related psychological domains and explore additional cultural settings.

**Supplementary Information:**

The online version contains supplementary material available at 10.1007/s00787-024-02506-7.

## Introduction

Attention deficit/hyperactivity disorder (ADHD) is a well-established neurodevelopmental condition characterized by age inappropriate and impairing levels of inattentiveness and/or impulsivity/hyperactivity [1, 2]. ADHD is generally considered to manifest similarly across cultures [3–5], with meta-analyses suggesting comparable prevalence rates [6, 7]. However, judgements about what level of behaviour constitutes a symptom, and therefore, who meets criteria for a diagnosis, vary considerably from one culture to another depending on adults’ sensitivity to children’s ADHD behaviours and standards of expected conduct [8–11]. This can be seen in a recent study comparing parents’ ADHD rating thresholds in Hong Kong and the United Kingdom (UK), cultures with contrasting child-rearing practices. Despite objectively higher activity levels in UK children, parents in Hong Kong rated their children higher on ADHD symptoms. This suggests potential cultural relativity in ADHD rating thresholds [12]. The study did not explore whether these differences impact the clinical meaning of the diagnosis. It remains unclear if lower activity levels could be of greater clinical significance in Hong Kong compared to the UK.

A growing number of studies give support to the view that ADHD is better conceptualised as a continuum than as a categorical ‘disorder’ [13–16]. This study extends this framework to analyse cross-cultural differences in the psychological correlates of ADHD symptom levels within community samples from HK and UK. This approach is important as applying the ADHD construct across cultures, particularly beyond Western societies, implicitly assumes it reflects the same underlying factors and mediating psychological processes in different cultural settings. Indirect comparisons of studies examining ADHD neuropsychological correlates in Eastern and Western cultures support this assumption. For instance, research on ADHD and executive function in Asian countries [17–20] reveals similar ADHD-related deficits in working memory, self-control, planning and attention shift tasks as seen in Western-based meta-analyses [21, 22]. However, direct cross-cultural comparisons to test this assumption of cultural invariance are rare. A more recent study involving children with ADHD from Japan and New Zealand found similar patterns of associations between ADHD symptom levels and behavioural sensitivity to punishment across both samples [23–25].

In this paper, we test the assumption of cultural invariance with regard to the ability of preschool children to wait for future events and outcomes. Waiting ability, a critical early marker of self-regulation, reflect a child’s capacity to manage behaviour and emotions in pursuit of long-term goals, rewards, or social expectations [26–29]. Developed in the preschool years and maturing throughout childhood [30–35], early individual differences in waiting predict future socio-emotional development, academic achievement, mental well-being and self-efficacy [27, 33, 36–38].

Delay aversion, a strong intolerance to delay, has been hypothesized as one of several different neuropsychological pathways to ADHD symptoms [39–41]. This suggests that symptoms of ADHD could be understood as a motivation to reduce or avoid the experience of delay when waiting for valued outcomes or important events [42, 43]. When delay is unavoidable, inattention and hyperactivity might serve to subjectively shorten the perceived wait duration [44, 45]. It has been consistently found that compared to typically development peers, children with ADHD exhibit greater difficulty inhibiting impulses when waiting for a ‘forbidden’ reward [46, 47]. Further, their levels of inattentiveness and hyperactivity increase with delay duration, suggesting a linear relationship between ADHD symptom and delay aversion [48, 49]. These findings support the hypothesis that individuals with higher levels of ADHD symptoms are more sensitive to delay and perform less optimally in waiting situations. Meta-analyses reveal medium-to-large sizes for these effects [47, 50, 51], even in preschool community samples [52, 53].

Waiting behaviours and abilities are likely to be shaped by cultural factors [54–56]. Eastern cultures, influenced by Confucianism and collectivism, emphasize self-regulation. Adults, including parents and teachers, hold high expectations for children’s behaviours [57–59]. Children are trained to exercise self-discipline and self-regulation [37, 60–64]. In contrast, Western cultures exhibit more relaxed parenting styles and wider acceptance of a broader range of conduct [65, 66]. These cultural differences, between East and West, seem especially relevant to the study of self-regulation [8, 10, 11]. In a cross-cultural study that directly and specifically comparing children’s waiting-related behaviours in Western and Eastern cultures, Ding et al. found that Chinese children exhibited greater willingness to wait for larger-but-delayed rewards compared to British children [67]. Interestingly, Chinese parents reported poorer inhibition in their children compared to parents in Sweden, Spain and Iran [68] – potentially reflecting higher expectations for children’s self-regulatory behaviours.

Despite cultural variations in expressing and reporting ADHD-related behaviours, previous indirect comparisons suggest similar correlations between ADHD symptoms and waiting responses in HK and UK [53, 69]. To the best of our knowledge, there has been no cross-cultural studies directly comparing the relationship between waiting behaviours and ADHD symptoms across Western and Eastern cultures. The study aims to to explore the cultural difference in the relationship between preschool ADHD symptoms and waiting-related performance and reactions through a direct comparison of children in HK and the UK. Building on existing literature from different nations, we hypothesized that –.

(i) children with higher levels of ADHD symptoms would show less adaptive waiting-related performance (e.g. lower tendency to wait and a greater emotional negativity during waiting).
(ii) the associations between ADHD and waiting-related performance would be similar in the UK and HK settings.

Because wating-related responses occur across a range of different settings and take a number of forms, we employed three tasks to measure different waiting elements and recorded outcomes in a number of different ways. The three tasks measured: *willingness/ability to wait for rewards, choosing the amount of time to wait, and frustration when having to wait unexpectedly when a task is interrupted*. In terms of outcomes, in addition to task performance we looked at participants’ waiting-related behavioural agitation (i.e., squirming and fidgeting) and negative emotional reactions (i.e., expression of frustrations).

## Methods

### Participants

One-hundred-and-eighty-nine preschool children (age in months: *M* = 46.22, *SD* = 5.73, range = 36.92–59.24) and their parents living in London UK (*n* = 68; 51% male) and HK (*n* = 121; 58% male) were recruited via local nurseries, preschools and online parent groups using social media adverts. Written informed consent had been appropriately obtained from parents. Inclusion criteria were age (36 to 60 months), estimated IQ of at least 80 and no diagnosis of neuropsychological disorder. Based on the results of the screening questionnaire completed by teachers and parents, thirty children (*n*^*UK*^=13 and *n*^*HK*^=17) were excluded on the following criteria— outside the age range; with IQ below 80; with special educational needs and/or a diagnosis of a pervasive developmental disorder (e.g., autism spectrum disorder); teacher non-engagement; family not able to attend testing sessions. No participants had been formally diagnosed with ADHD and none was taking ADHD medications.

To ensure we included participants with a range of levels of ADHD symptoms and that we compared like with like across cultures, children were screened using the five-item hyperactivity/ inattention subscale of *The Strengths and Difficulties Questionnaire* completed by parents and teachers (SDQ, version T2-4) [70]. During the initial stage of recruitment, we encountered a skew towards participants with medium/ high SDQ ratings. To address this imbalance and achieve statistically comparable groups in terms of ADHD symptoms, i.e. that the HK and UK samples had similar proportions of participants with low or medium/high ADHD symptoms, we employed oversampling followed by random exclusion in the oversampled groups. The number of children having elevated levels of ADHD symptoms rated by parents **or** teachers (subscale score ≥ 5) in the final UK and HK samples was not statistically different, *χ*^*2*^*(*1) = 1.27, *p* = .26. The average subscale scores in the final UK and HK sample were not statistically different, *F*(1, 110) = 2.33, *p* = .130. To achieve at least 80% statistical power and a medium effective size (correlation coefficient *r* = .3) with significance level of 0.05, it was estimated that a sample of 80 participants was needed. Full data was available for 112 children (*n*^UK^=55 and *n*^HK^=57; females = 49 and males = 63; mean age = 46.20 months, *S*.*D*. = 5.73; range = 36.92–59.24).

This study was conducted as part of a larger longitudinal investigation. After a period of 12 months, participants were followed up with questionnaires, and a subset of participants also repeated the waiting tasks. Test-retest reliabilities for the rating scales and waiting tasks were established using data from this follow-up assessment.

### Measures

#### Screening measures

**Inattentive and overactive behaviours screener**. The parent and teacher versions of the *Strengths and Difficulties Questionnaire* (SDQ, version T2-4) are widely used psychometrically strong, brief screening questionnaires designed for research/clinical purposes [70]. The hyperactivity/inattention subscale consists of five items: two measuring inattention, two hyperactivity and one impulsivity. The original English language version was used in the UK. A validated Chinese translation was used in HK [71]. We opted for the brief 5-item SDQ subscale to minimize burden on teachers and parents during the initial screening stage, and to address the challenges of recruiting teachers with heavy workloads.

**Intelligence**. Children’s IQ was estimated using the Block Design and Vocabulary subtests of *Wechsler Preschool and Primary Scale of Intelligence* (WPPSI-III) [72]. The WPPSI measures the cognitive ability of preschoolers and young children between 2 years 6 months and 7 years and 3 months. The estimated IQ was derived from the WPPSI-III conversation table (Table B-14) using the sum of the scaled scores from the two subtests [73]. The English (UK) and Traditional Chinese language versions were used in the UK and HK respectively.

#### Baseline activity level and affect

Children’s baseline activity level was measured during a period of unstructured free play that lasted eight minutes using an unobtrusive wrist-worn triaxial actigraph unit accelerometer. Before the start of the free play, children were informed that they could choose any activity or toys available. The activity tracker measured the *g*-force (*g*) at 6.5 Hz over and provided an average *g* per 1 s epoch. A higher average *g* indicated a higher activity level.

To control for potential influences, children’s general affect during free play was coded using the positive affect (smiling, laughing) and negative affect (frowning, cold/harsh voice) codes from the Parent-Child Interaction System (PARCHISY) [74, 75]. The negative affect code was used as a controlled variable in the analysis.

#### Waiting tasks

We employed three waiting tasks that tapped participant’s performance and their behavioural and emotional reactions to different waiting situations. Most previous studies only include performance indicators of waiting tolerance (e.g. choice of immediate reward, retrieval of prohibited reward before waiting time ends), but it has been shown that individuals with higher levels of ADHD symptoms also display more intense emotional responses during delay [49, 76, 77]. Therefore, in each task, waiting-related behavioural agitation (i.e., squirming/ fidgeting) and negative emotional reactions (i.e., frustration as indicated by frowning, sighing and pouting) were observed and coded by trained researchers (two in each university) using a 4-point scale with – 1=“None/ Very rare − 0–10% of time”, 2=“A little − 11–25% of time”; 3=“Quite a lot − 25–50% of time” and 4=“A lot - >50% of time”. A higher score reflects a higher level of waiting-related agitation and frustrations.

In both UK and HK, coders were trained on five videos taken from the pilot trials, where the focus was on testing the logistics of the protocol, task administration, and scoring procedures. The coders achieved a minimum of 90% agreement on their ratings before starting the official video coding. The pilot trial videos were used solely for training purposes and were excluded from the final data analysis. For the official video coding, we employed a double-coding approach to ensure reliability in our data analysis. This involved having at least two independent coders analysed each video. To assess the agreement between coders, we calculated intraclass correlation coefficients (ICCs) for both behavioural and affective codes for all the videos used in the final analysis. The ICCs were excellent, with values of 0.98 and 0.95 respectively, indicating a very high level of consistency between coders.

**Willingness/ability to wait for rewards.** An adapted version of the Cookie Delay Task (CDT) was developed to measure children’s ability to delay gratification and to gauge their reactions during the waiting period [78, 79]. The original task involved hiding an edible treat under one of the three containers and instructing participants to wait for a signal until they were allowed to retrieve it. In this study, as in other recent studies, attractive stickers were used instead of cookies. To make waiting more motivating, children were presented with an appealing bonus sticker, which could be either sparkly or 3D. They were then informed that they could earn the bonus sticker by waiting patiently until the researcher clapped their hands, signalling to retrieve the hidden sticker. This adapted version included eight trials with varying delay intervals between 5-sec and 40-sec. Children’s willingness/ability to wait for rewards was coded based on their behaviour, with 2 = “not inhibited” (found and retrieved the reward before the signal); 1 = “partially inhibited” (touched the cup but did not take the reward); and 0 = “fully inhibited” (waited until the signal is given) [53]. An average inhibition score was computed by adding scores of all the trials, with a higher score indicating a lower level of inhibition. In this sample, Cronbach’s *α* for the eight trials was 0.72 and test-retest reliability was 0.87.

**Choosing the amount of time to wait.** The Bee Delay Task (BDT) was specifically designed to be developmentally appropriate for preschoolers to measure their delay-related responses in terms of pre-emptively judging how long they would be able/willing to wait and then seeing if they did wait that long [80]. Children were shown seven flowers on a computer screen and told that a bee would go to each flower to collect nectar. They were also told that they would earn one point (which could be exchanged for stickers afterwards) for each flower that the bee landed on. It was also explained to them that the bee would get tired with each flower it landed on, and it would take longer for it to fly to the next flower (i.e., the amount of delay between flowers would increase with a rate of 125% per flower). They were asked to choose the number of flowers they wanted their bee to visit before the trial started. Children were also told that they could press a button during a trial to stop the bee before their chosen number of flowers, if they preferred (i.e., terminate the trial earlier). If they pressed it, the trial ended immediately, and they would get the points they had won up to then. There was a total of 10 trials. Children’s judgements about how long they would wait were indicated by the discrepancy between the number of flowers/waiting time chosen and the number of flowers/waiting time they actually experienced before they stopped the trial, with a higher average score indicated a higher level of early termination of the waiting task. In this sample, Cronbach’s *α* for the ten trials was 0.99 and test-retest reliability was 0.88.

**Having to wait unexpectedly when a task is interrupted.** The computerized Preschool Delay Frustration Task (P-DeFT) is a preschool version of a task created by Bitsakou et al. to measure children’s behavioural and affective expressions of frustration when a continuous presentation of a reinforced task is unexpectedly interrupted [48, 81, 82]. The P-DeFT was designed to be a simple and enjoyable “shopping” game [82]. During each trial of the game, participants were first shown a red *Wait* signal and then asked to complete a two-stage task – (1) press a “crossing” button to change the *Wait* signal to a green *Go-signal* then (2) visit a “toy supermarket” and get a target object as shown by the experimenter. The only rule of the game for participants was to wait for the red signal to turn green before proceeding to find the item. There was a total of 18 trials. In the majority of trials (*n* = 12), the *Go-signal* was shown immediately after the child pressed the crossing button (i.e. no *pre-Go-signal* delay). In six trials, presented in a pseudo-random order, a *pre-Go signal waiting period* (either 5-secs or 10-secs; three trials each) was imposed. Participants were not informed beforehand about the presence of these extra delay periods but were told that the crossing button was rather old and might occasionally be a bit slow to work. On tasks of this sort, participants’ frustration levels are operationalized by both the amount of button presses during the *pre-Go-signal* waiting period and by the amount of activity displayed in *post-delay* shopping task [82]. *Post-delay* activity was measured by the wrist-worn actigraph unit. A higher factor score comprised of the two variables indicated a higher level of delay-related frustration. In this sample, Cronbach’s *α* for the six trials was 0.88 for duration of presses and 0.91 for post-delay activity.

#### Rating scales

**ADHD symptom rating.** Parents rated their children on the 18 DSM-IV ADHD symptoms using the *ADHD Rating Scale IV* adapted for preschoolers (ADHD-RS-IV-P) [83, 84]. Informants rate the frequency of occurrence of the described symptom using a 4-point scale: 0=“Never or rarely”, 1=“Sometimes”, 2=“Often” and 3=“Very often”. A total score was computed by adding the scores of all the items. In this sample, Cronbach’s *α* for the full scale was 0.91 and test-retest reliability was 0.84.

**Delay aversion ratings.** Parents rated their children’s delay aversion using a 10-item Quick Delay Questionnaire (QDQ). The QDQ was originally designed to measure adults’ self-reported delay related behaviours [85]. It was adapted to be used to evaluate preschoolers’ delay related difficulties by teachers or parents [80]. The ten items (e.g. “Hates waiting for things”, “Often gives up on things he or she can’t have immediately”) were rated against a 5-point Likert scale from 1=“Not at all like him/her” to 5=“Very much like him/her”. An average score was computed by dividing the sum of item scores by ten. The scales have high internal consistency (*α*s ≥ 0.84) and good test-retest reliability (*r*s ≥ 0.75).

#### Procedures

Test sessions took place in quiet rooms either at King’s College London or the University of Hong Kong. Mother-child dyads were briefed that this was a cross-cultural study exploring preschoolers’ behaviours in tasks that require sustained attention, patience and waiting. Comprehensive information regarding the participants’ rights, data protection and confidentiality was provided to parents prior to consent being sought. Researchers then explained to the participating children in standardized simple terms, with age-appropriate materials, what was going to happen in a session and their rights to stop doing the tasks at any time. Children indicated whether they wanted to take part in the project by circling their preference (“yes” or “no”). After the introduction, the children had a free play session with their mothers and then completed the waiting tasks (in the order of CDT, BDT then P-DeFT)administered by trained researchers (one each in UK and HK) while their parents filled in questionnaires in a separate room. The complete administration of CDT, BDT and P-DeFT lasted on average six, seven and eight minutes respectively. Breaks were taken between tasks. In line with the BPS guidelines emphasizing the importance of children’s rights to withdraw from the study at any time, researchers regularly monitored children’s assent by sensitively attending to any signs, verbal or nonverbal, that they were no longer wholly willing to continue with the data collection. The research team presented a certificate and a book voucher valued at £5 or HK$50 to each participating parent-child pair as a token of appreciation.

### Data analysis

#### Preparatory analysis

A small proportion of the CDT (3%), BDT (6%) and P-DeFT (1%) programme data and actometer reading data (4%) were missing due to technical issues and participants’ withdrawal from particular tasks. Where data were missing, we used pairwise deletion to optimize data availability.

As a range of tasks were used to examine children’s waiting-related behaviours, there were a relatively large number of variables. To reduce the number of variables and potential for multiple testing, we conducted exploratory factor analysis using the SPSS to identify underlying dimensions in the task performance and behavioural and emotional reactions across the three waiting tasks. Factor scores were derived by the SPSS using the regression method, i.e. regressing each original variable on the extracted factors. The resulting factor scores represented the estimated level of each participant on the latent factor and were employed in subsequent analyses.

After analysing and comparing the demographic characteristics of the UK and HK samples, we then examined if there were age, sex, IQ and family income effects on the delay aversion ratings and waiting-related measures using analysis of variance (ANOVA), chi-square test and correlational analyses. Corrections for multiple testing were made using Bonferroni formula. Confounding variables, if any, were controlled for in the subsequent analyses.

#### Core analysis

Pearson’s correlations were used to examine the relationship between participants’ ADHD symptom levels, delay aversion ratings, waiting task performance and waiting-related reactions. We then compared the coefficients of the correlation between ADHD symptoms, delay aversion and waiting-related responses in the UK and HK samples using Fisher’s Z-transformation. The PROCESS macro test of moderation developed by Hayes [86] using SPSS (model 1, 5000 bootstrap samples) was subsequently conducted to explore whether the relationship between ADHD symptom ratings and waiting-related measures was moderated by national group.

## Results

Table 1 presents the demographics and background characteristics of the sample. UK and HK participants did not differ significantly in age, estimated IQ, SDQ hyperactivity/inattention subscale rating, baseline activity level and affect, sex ratio, household composition and income.

**Table 1:** Characteristics of child participants in UK and HK

		UK (*n* = 55)				HK (*n* = 57)				
		M	SD	Range		M	SD	Range		Statistical comparison
Age (months)		46.55	6.49	37–59		45.86	4.91	37–54		*F*(1, 110) = 0.41, *p* = .526
Estimated IQ		108.72	12.20	82–132		105.26	10.69	82–124		*F*(1, 109) = 2.53, *p* = .114
SDQ hyperactivity/inattention subscale rating		3.50	2.07	0–10		4.04	1.69	1–7		*F*(1, 110) = 2.33, *p* = .130
Baseline activity level		203.22	43.45	130–320		196.96	46.15	72–302		*F*(1,105) = 0.52, *p* = .472
Baseline negative affect		1.17	0.43	1–3		1.05	0.23	1–2		*F*(1,83) = 2.29, *p* = .134
Female – *n* (%)		25 (45.45)				24 (42.11)				*χ²* (1, 112) = 0.13, *p* = .721
Living with both parents – *n* (%)		51 (92.7)				55 (96.5)				*χ²* (1, 112) = 0.78, *p* = .376
Monthly household income – *n* (%)										
Below £2000		4 (7.27)				6 (10.53)				*χ²* (3, 112) = 5.33, *p* = .149
£2000–2999		1 (1.82)				7 (12.28)				
£3000–3999		8 (14.55)				8 (14.04)				
Above £4000		42 (76.36)				36 (63.16)				

Table 2 shows the intercorrelations between the waiting-related measures from the three tasks. In each waiting task, there were positive correlations found between children’s task performance and their observed behavioural and emotional reactions. Between tasks, although the nature of the waiting required in the three tasks is different, participants’ performance and responses on these tasks were intercorrelated, which is consistent with prior studies [49, 76, 77, 80].

**Table 2:** Intercorrelations between waiting task performance, observed waiting-related behavioural agitation and negative affect

			1	2	3	4	5	6	7	8
1	Waiting task performance	BDT								
2		CDT	0.25*							
3		P-DeFT	0.27*	0.17						
4	Waiting-related behavioural agitation	BDT	0.22	0.25*	0.33**					
5		CDT	0.32*	0.44**	0.54**	0.47**				
6		P-DeFT	0.34**	0.36**	0.49**	0.31*	0.60**			
7	Waiting-related negative affect	BDT	0.20	0.21	0.17	0.43**	0.50**	0.31*		
8		CDT	0.17	0.46**	0.35**	0.39**	0.55**	0.37**	0.44**	
9		P-DeFT	0.28*	0.36**	0.36**	0.49**	0.50**	0.42**	0.54**	0.54**

Note: BDT = Bee Delay Task; CDT = Cookie Delay Task; P-DeFT = Preschool Delay Frustration Task. * *p* < .02; ** *p* < .001 (adjusted *p* values based on Bonferroni correction)

Performance scores on the BDT, CDT and P-DeFT were positively correlated (*r*s ≥ 0.17; *p*s ≤ 0.080). Factor analyses revealed a single latent factor underlying these three variables, explaining 48.6% of the variance (supplementary table S1). We labelled this factor as *waiting task performance*, with higher factor scores indicating lower levels of inhibition and waiting tolerance during the waiting tasks.

The observed waiting-related behavioural agitation and negative affect from the three tasks were positively correlated with each other (*r*s ≥ 0.31, *p*s ≤ 0.001 and *r*s ≥ 0.44, *p*s < 0.001 respectively). Factor analyses revealed a single latent factor underlying all these six reaction codes which explained 54.5% of the variance (supplementary table S2). This factor was labelled *waiting-related behaviours and reactions*. Factor scores were computed using SPSS, with higher values indicating higher levels of waiting-related agitation and frustration.

Exploration of potential covariates using ANOVAs revealed no significant differences in any ratings or outcome variables between male and female participants (*Fs* ≤ 2.54, *ps* ≥ 0.114) or household income groups (*Fs* ≤ 1.33, *ps* ≥ 0.270) (supplementary table S3). Similarly, correlational analyses showed no significant associations between participants’ IQ, age, and any ratings or waiting-related variables (*rs* ≤ 0.23, *ps* ≥ 0.020). Consequently, these variables were excluded as covariates in subsequent analyses (supplementary table S4).

Table 3 presents the UK and HK participants’ ADHD symptom and delay aversion ratings as well as their waiting-related performance and reactions. HK children were rated by their parents as having higher levels of ADHD symptoms than UK children, while UK children displayed more intense waiting-related behaviours and reactions than HK children.

**Table 3:** ADHD symptom levels, delay aversion and waiting-related responses of participants in UK and HK

		UK					HK					Statistical comparison		
		M	SD	Range	*n*		M	SD	Range	*n*		*F*	*p*	*η* ^2^
ADHD symptom ratings		19.13	9.05	3–47	55		23.33	9.68	7–40	57		5.64	0.019	0.05
Delay aversion ratings		2.84	0.70	1.4–4.8	55		2.94	0.48	2.0-4.2	57		0.78	0.378	0.01
Waiting task performance		0.03	1.20	-1.6-2.8	47		− 0.02	0.80	-1.6-2.3	55		0.06	0.804	0.00
Waiting-related behaviors and reactions		0.31	1.21	-1.3-2.7	47		− 0.27	0.69	-1.3-1.7	55		9.06	0.003	0.08

Table 4 shows that the correlations between participants’ ADHD symptom levels, delay aversion and waiting-related responses were significant, suggesting that children with higher levels of ADHD symptoms showed lower level of tolerance and higher level of agitation and frustrations during the waiting tasks. The effect sizes of the correlation between the two ratings and waiting-related measures were medium (*rs* ≥ 0.30, *p* ≤ .002), while the correlation between ADHD symptom and delay aversion ratings was strong (*r* = .63, *p* < .001). The correlations remained significant with medium-to-large effect sizes, even after controlling for children’s baseline activity level and negative affect observed during the non-waiting context (free play) (supplementary table S5).

**Table 4:** Correlations between ADHD symptom levels, delay aversion and waiting-related responses

		1				2				3		
		Whole	UK	HK		Whole	UK	HK		Whole	UK	HK
1	ADHD symptom ratings											
2	Delay aversion ratings	0.63**	0.72**	0.55**								
3	Waiting task performance	0.38**	0.33	0.51**		0.37**	0.32	0.47**				
4	Waiting-related behaviours and reactions	0.30*	0.42*	0.50**		0.38**	0.46**	0.46**		0.62**	0.69**	0.55**

Note: * *p* < .01; ** *p* < .001 (adjusted *p* values based on Bonferroni correction)

Split-sample cross-sectional correlational analyses were conducted (Table 4), and the correlation coefficients between ADHD symptom levels, delay aversion and waiting-related responses in the two samples were compared using Fisher’s *Z*-transformation. The *Z*-tests showed that there were no significant differences between the correlation coefficients in the UK and HK samples (*Zs* ≤ 1.51, *p* ≥ .131). Despite the national group differences in ADHD symptom ratings and waiting-related behaviours and reactions, there was no significant between-nation differences in the associations between ADHD symptom levels and waiting-related responses. This was further explored in a PROCESS macro test of moderation which showed that the relationship between ADHD symptom ratings and waiting-related measures was not significantly moderated by national status. The main effect of ADHD symptom ratings on children’s waiting-related reactions was significant (*t* = 2.61, *p* = .010), but the interaction between ADHD symptoms and national group was not significant (*t* = 1.34, *p* = .184), suggesting a similar association between ADHD symptom ratings and waiting-related reactions across national groups. Similarly, the interaction effect between ADHD symptoms and national group on children’s waiting task performance was also not significant (*t* = 0.33, *p* = .743).

## Discussion

Delay aversion theory proposes that children with ADHD find waiting particularly difficult [39, 42, 43]. While research has provided support for this in Western cultures [47, 50–53], generalizability to other cultural contexts remains unexplored. The current research is the first study to explore the association between ADHD symptoms and waiting-related responses in non-clinical samples in two cultures known to have different expectations for children’s behaviour and self-regulation, UK and HK. There were a number of findings of note.

First, our analysis revealed medium to strong associations between parent-rated ADHD symptom and the two aggregated delay-related measures, even after controlling for children’s baseline activity level and negative affect during free play. Children with higher levels of ADHD symptoms displayed lower levels of inhibition and exhibited greater waiting-related agitation and frustration. These findings align with previous research showing that children with ADHD are more likely to act prematurely for a forbidden reward [47, 52] and exhibited more intense negative behaviours and emotions while waiting [48, 49, 81, 87, 88]. This supports the delay aversion hypothesis proposed by Sonuga-Barke that individuals with elevated ADHD symptoms find pre-reward delay emotionally aversive and may attempt to avoid or escape it [39, 42, 43].

Second, it was found that the associations between ADHD symptom severity and delay-related behaviours and reactions were similar in the HK and UK samples. Our findings align with prior research suggesting cultural differences in parent rating threshold of ADHD symptoms [12]. While UK children displayed greater waiting-related agitation, parents in HK reported higher ADHD symptoms in their children [12]. Nevertheless, our cross-cultural comparison revealed no statistically significant interaction between national group and the association between ADHD symptoms, delay aversion and waiting-related responses. This suggests that within each country, ADHD symptoms correlate similarly with waiting-related performance and responses. In this regard, ADHD symptoms appear to exhibit a degree of consistency in behavioural manifestations and psychological characteristics across cultures. This is important to know the cross-cultural meaning of ADHD symptoms in terms of their psychological correlates because in HK and many other Asian cultures, the early identification and intervention efforts for children at risk of ADHD rely heavily on Western-based literature, which assumes ADHD is a universal construct [10, 89]. While the current findings demonstrate similar patterns of associations between ADHD symptoms and delay-related constructs in both HK and UK samples, we have to be cautious not to assume that the ADHD and delay aversion constructs are culturally invariant based solely on this study of a community sample. Further research can extend this cross-cultural analysis to other ADHD-related psychological domains across diverse nations, including both clinical and non-clinical samples.

### Strengths

This study has several strengths. First, it employed a comprehensive battery of established and novel tasks that assessed preschool children’s waiting-related performance and responses across different waiting durations and nature. This approach goes beyond relying on a single measure. Second, the study utilized objective performance measures alongside coded observations of participants’ behavioural and emotional reactions during waiting periods, providing a richer picture of waiting behaviour. Third, the relatively large, culturally diverse sample recruited from two populations known for distinct child-rearing practices offers valuable insights into the generalizability of delay aversion theory across cultures.

### Limitations

This study also has limitations. First, we attempted to match participants in the UK and HK samples based on parent and teacher ratings of ADHD symptoms using the SDQ. However, as our analyses have now shown that parents applied different ADHD rating thresholds [12], in hindsight, matching based on objectively measured activity might have been more appropriate. Second, this study lacked a non-waiting but frustration-provoking task that allows us to differentiate between frustration specific to waiting situations and a more general propensity for emotional and behavioural displays of frustration. For instance, including a task with high difficulty or frequent losses could help clarify whether the observed behavioural agitation and emotional expressivity in children with ADHD are specific to waiting contexts. Third, the sample lacked a significant number of participants with very high ADHD symptom levels. This limited our power to investigate associations between ADHD symptoms, delay aversion, and waiting task responses within the clinical range. Lastly, stickers were used as rewards in the waiting tasks. Such an approach has been used successfully in previous studies with young children [90, 91]. In our study, participants were offered a selection of three sticker types (e.g., dinosaurs, princesses, cars, cats, etc.) to choose from prior to the start of each waiting game. All participants were observed to exhibit high engagement with the provided sticker choices, suggesting a positive response to the reward system. The possibility remains that employing primary rewards, such as snacks or playtime, could yield different results.

### Future direction

Our findings suggest several important directions for future research. First, the current study employed waiting tasks with a limited number of trials and waiting time variations due to the participants’ young age. Future studies with older participants could utilize longer delay intervals to examine how waiting-related responses change in magnitude and direction as delay length increases and whether the association between ADHD symptoms and aversive waiting reactions varies with delay duration. Second, recruiting samples with a broader representation of ADHD severity would strengthen the generalizability of findings, particularly regarding associations within the clinical range. Finally, employing larger sample sizes would facilitate more comprehensive statistical analyses, including robust factor analyses to explore potential underlying latent constructs related to ADHD symptoms and waiting behaviours.

### Clinical implications

There are a number of potential clinical implications of our findings. First, this study found a significant correlation between ADHD symptoms and the performance and reactions of preschool children during waiting tasks. While longitudinal clinical studies are needed to determine if these waiting-related responses predict future ADHD diagnoses, these results suggest that measuring preschoolers’ emotional and behavioural reactions to waiting could be a potentially valuable tool in understanding ADHD-related psychological aspects. Second, the between-nation difference found in the parent ratings and waiting-related responses suggests the need to apply a different cultural threshold when conceptualizing and assessing early waiting-related difficulties, while at the same time recognizing that common processes appear to drive the relationship between ADHD ratings and waiting-related responses in both cultures. Third, the common associations found between ADHD symptoms and delay aversion may provide insights for early intervention efforts. For instance, there could be potential value in incorporating a delay-related focus into those parent training programmes, currently recommended for children at risk of ADHD under five years as first-line treatment [92, 93]. Parents and children could be encouraged to practice strategies like self-directed speech and distraction techniques to better deal with delay and its associated frustrations [87]. Further, randomized control trials are needed to explore the efficacy of interventions designed to encourage waiting behaviours in children with ADHD in terms of core symptom reduction.

In summary, parent ratings of ADHD symptoms were associated with preschoolers’ waiting-related performance, negative emotions and behavioural agitation in both HK and the UK. The results highlight the cross-cultural commonalities in the neuropsychological correlates of ADHD with regard to waiting, despite the between-nation differences in rating threshold and waiting-related responses in absolute terms.

## Electronic supplementary material

Below is the link to the electronic supplementary material.

Supplementary Material 1

## Data Availability

No datasets were generated or analysed during the current study.
