# Supplementary material for: Attention-deficit/hyperactivity disorder (ADHD) in cultural context II: a comparison of the links between ADHD symptoms and waiting-related responses in Hong Kong and UK
Source: Eur Child Adolesc Psychiatry. 2024 Jun 27;34(2):633–45. doi: 10.1007/s00787-024-02506-7 (PMC11868378; doi:10.1007/s00787-024-02506-7)
Supplement: Supplementary file 1 — Supplementary Material 1 [file 787_2024_2506_MOESM1_ESM.docx]

**Supplementary materials**

**Observation coding**

Participants’ waiting-related frustration as expressed in terms of behavioural agitation and negative affect were coded for each of the delay trials in the three waiting tasks by two independent coders. The coders were trained using training video recordings taken during the pilot stage and achieved a minimum of 90% agreement on their ratings before starting the official video coding.

The coders looked for specific cues of frustration, categorized into two main types: (1) **Behavioural Agitation** and (2) **Negative Affect**: frowning, sighing and pouting. The coders were instructed to consider the wait duration when assigning a rating using a 4-point scale to each observed cue. To ensure consistency between coders, inter-rater reliability was assessed, with intraclass correlations (ICCs) for the behavioural agitation and negative affect codes as .92 or higher (average = .95) and .95 or higher (average = .95) respectively. The coding system is as follow:

Instructions:

- Coders independently rate participants’ waiting-related frustration based on observed behaviours and emotional expressions during each delay trial within each task.
- Ratings will be based on the duration of displayed behaviours throughout the delay period, which may vary across trials and tasks. For instance, a 10-second instance of squirming during a 1-minute wait would be coded as “1” (17%), while the same duration of squirming during a 30-second wait would be “2” (33%).

Coding Categories:

1. Physical Reaction to Delay/ Behavioural Agitation. Examples include squirming in the chair, shifting body weight, fidgeting with hands or objects, tapping feet.
2. Not at all/Rare (0-10% of time): No or rare fidgeting or squirming observed.
3. A Little (11-25% of time): Occasional fidgeting or squirming.
4. Quite a Lot (25-50% of time): Frequent fidgeting or squirming.
5. A Lot (>50% of time): Constant fidgeting or squirming.
6. Emotional Reaction to Delay/ Negative Affect. Examples include facial expressions like frowning, grimacing and pouting, as well as vocalisations like signing, whining, grunting and verbal complaints about waiting.
7. Not at all/Rare (0-10% of time): No or rare signs of frustration displayed.
8. A Little (11-25% of time): Occasional facial expressions, verbal or non-verbal cues of frustration.
9. Quite a Lot (25-50% of time): Frequent facial expressions, verbal or non-verbal cues of frustration.
10. A Lot (>50% of time): Consistent expressions of frustration throughout the delay period (e.g., continuous frowning, sighing, and verbal complaints).

Table S1. Exploratory factor analysis for the performance scores on the waiting tasks

| **Measure** | **Factor loadings** |
| --- | --- |
| BDT performance | .731 |
| P-DeFT performance | .680 |
| CDT performance | .678 |
|  |  |
| **Eigenvalue** | 1.46 |
| **Variance (%)** | 48.57 |
| KMO = .60; Bartlett’s Test of Sphericity = 14.35 (*df* = 3, *sig* = .002). | |

Table S2. Exploratory factor analysis for the performance scores on the waiting tasks

| **Measure** | **Factor loadings** |
| --- | --- |
| CDT waiting-related behavioural agitation | .818 |
| P-DeFT waiting-related negative affect | .792 |
| CDT waiting-related negative affect | .748 |
| BDT waiting-related negative affect | .738 |
| BDT waiting-related behavioural agitation | .685 |
| P-DeFT waiting-related behavioural agitation | .649 |
|  |  |
| **Eigenvalue** | 3.29 |
| **Variance (%)** | 54.54 |
| KMO = .83; Bartlett’s Test of Sphericity = 207.08 (*df* = 15, *sig* < .001). | |

Table S3. Sex and household income group differences in ADHD and delay aversion ratings and waiting task measures

|  |  | **Sex difference** | **Household income difference** |
| --- | --- | --- | --- |
| 1 | ADHD symptom ratings | *F* ^a =^ 2.26, *p* = .135 | *F* ^c^ = .75, *p* = .522 |
| 2 | Delay aversion ratings | *F* ^a =^ 2.54, *p* = .114 | *F* ^c^ = .30, *p* = .825 |
| 3 | Waiting task performance | *F* ^b =^ 1.59, *p* = .210 | *F* ^d^ = 1.33, *p* = .270 |
| 4 | Waiting-related behaviours and reactions | *F* ^b =^ .42, *p* = .519 | *F* ^d^ = .82, *p* = .484 |

Note. The *df* of variables for *t* statistics are *a* = (1, 110); *b* = (1, 100); *c* = (3, 108); *d* = (3, 98) respectively.

**Table S4. Correlations between IQ, age, ADHD and delay aversion ratings and waiting task measures**

|  |  |  | **IQ** | **Age** |
| --- | --- | --- | --- | --- |
| 1 | ADHD symptom ratings | | -.21 | -.08 |
| 2 | Delay aversion ratings | | -.15 | .08 |
| 3 | Waiting task performance | | -.21 | -.23 |
| 4 | Waiting-related behaviours and reactions | | .00 | -.03 |

Note: * *p* < .01; ** *p* < .001 (adjusted *p* values based on Bonferroni correction).


**Table S5 Partial correlations between ADHD symptom levels, delay aversion and waiting-related responses, controlling for baseline activity and negative affect**

|  |  | 1 | 2 | 3 |
| --- | --- | --- | --- | --- |
| 1 | ADHD symptom ratings |  |  |  |
| 2 | Delay aversion ratings | .63** |  |  |
| 3 | Waiting task performance | .39** | .34* |  |
| 4 | Waiting-related behaviours and reactions | .30* | .35* | .58** |

Note: * *p* < .01; ** *p* < .001 (adjusted *p* values based on Bonferroni correction).
